# Exogenous Shocks and Business Process Management: A Scholars’ Perspective on Challenges and Opportunities

**DOI:** 10.1007/s12599-021-00740-w

**Published:** 2022-02-10

**Authors:** Maximilian Röglinger, Ralf Plattfaut, Vincent Borghoff, Georgi Kerpedzhiev, Jörg Becker, Daniel Beverungen, Jan vom Brocke, Amy Van Looy, Adela del-Río-Ortega, Stefanie Rinderle-Ma, Michael Rosemann, Flavia Maria Santoro, Peter Trkman

**Affiliations:** 1 FIM Research Center, Project Group Business & Information Systems Engineering of the Fraunhofer FIT, University of Bayreuth, Wittelsbacherring 10, 95444 Bayreuth, Germany; 2 South Westphalia University of Applied Sciences, Soest, Germany; 3 Project Group Business & Information Systems Engineering of the Fraunhofer FIT, Bayreuth, Germany; 4 European Research Center for Information Systems (ERCIS), University of Münster, Münster, Germany; 5 Paderborn University, Paderborn, Germany; 6 University of Liechtenstein, Vaduz, Liechtenstein; 7 Gent University, Gent, Belgium; 8 University of Seville, Seville, Spain; 9 University of Vienna, Vienna, Austria; 10 Queensland University of Technology, Brisbane, Australia; 11 State University of Rio de Janeiro, Rio de Janeiro, Brazil; 12 University of Ljubljana, Ljubljana, Slovenia

**Keywords:** Business process management, Exogenous shocks, Challenges, Opportunities

## Abstract

Business process management (BPM) drives corporate success through effective and efficient processes. In recent decades, knowledge has been accumulated regarding the identification, discovery, analysis, design, implementation, and monitoring of business processes. This includes methods and tools for tackling various kinds of process change such as continuous process improvement, process reengineering, process innovation, and process drift. However, exogenous shocks, which lead to unintentional and radical process change, have been neglected in BPM research although they severely affect an organization’s context, strategy, and business processes. This research note conceptualizes the interplay of exogenous shocks and BPM in terms of the effects that such shocks can have on organizations’ overall process performance over time. On this foundation, related challenges and opportunities for BPM via several rounds of idea generation and consolidation within a diverse team of BPM scholars are identified. The paper discusses findings in light of extant literature from BPM and related disciplines, as well as present avenues for future (BPM) research to invigorate the academic discourse on the topic.

## Introduction

The COVID-19 pandemic has changed our daily lives since early 2020. Apart from its medical implications and humanitarian costs, the pandemic has had a profound effect on the global economy (Chakraborty and Maity 2020). Organizations have experienced disruptive changes, not only regarding their internal operations but also their interactions with their environment. The pandemic has led to a dramatic increase in employees working from home, hibernating organizations, the collapse of supply chains entailing the shutdown of production facilities and stores, digital rather than paper sign-off procedures, and fast-tracked innovation and product go-lives (Guan et al. 2020; Gruszczynski 2020; Seetharaman 2020). This induced dramatic changes in managerial and operational processes.

From an organizational perspective, the pandemic constitutes an exogenous shock – an unanticipated, low-likelihood event stemming from the external environment and entailing disruptive changes with potentially existence-threatening consequences (Taleb 2010). Despite its severity, the COVID-19 pandemic is not the only exogenous shock that organizations have had to tackle in recent years; for example, they also faced the 2008 global financial crisis (Roy and Kemme 2020), Brexit (Todd 2017), the US–China trade war (Thomas et al. 2020), and the Fukushima nuclear disaster (Wakiyama et al. 2014). While not all exogenous shocks have been, or will be, as severe as the COVID-19 pandemic, organizations will probably experience such events more frequently since the economy is becoming increasingly volatile, uncertain, complex, ambiguous, and hyperconnected (World Economic Forum 2016; Beverungen et al. 2020).

While the market-level effects of exogenous shocks have already been studied (Kilian 2008; Fridgen et al. 2015; Chakrabarti 2015; Li et al. 2016), we focus on their effects on individual organizations – specifically on business processes and business process management (BPM). As a corporate capability (Rosemann and vom Brocke 2015), BPM drives intentional process change, particularly continuous process improvement and business process reengineering (Hammer et al. 2015). It also aims to enhance organizations’ ability to cope with unintentional process change, both by preventing it through process compliance and by harnessing positive effects in terms of positive deviance (König et al. 2018). Unintentional process change encompasses process drift (Pentland et al. 2020; Beverungen 2014) and disruption (e.g., exogenous shocks) (Mendling et al. 2020). Since the latter affects organizations more severely than other kinds of process change, it is highly relevant to BPM researchers. Despite the presence of important works connected with crisis prevention and management, including organizational resilience and high-reliability organizations (Antunes and Mourão 2011; Salovaara et al. 2019), the intersection of exogenous shocks and BPM is neither well understood, nor do methods and tools for addressing associated challenges and opportunities exist. Against this backdrop, this research explores the following research question: *What challenges and opportunities exist for BPM due to exogenous shocks?*

To answer this research question, a diverse group of BPM scholars, each with close connections to industry and BPM practice, joined forces and co-authored this research note. After developing a common conceptualization of the interplay between BPM and exogenous shocks, we identified 24 challenges and opportunities for BPM structured according to the well-known six core elements of BPM (de Bruin and Rosemann 2007) through multiple rounds of idea generation and consolidation. We discuss these challenges and opportunities considering literature from BPM and disciplines related to crisis management. Our results aim to foster the understanding of the interplay between exogenous shocks and BPM and to guide future BPM research.

The remainder of this paper is organized as follows: In Sect. 2, we provide relevant background regarding exogenous shocks and BPM. After outlining our research approach in Sect. 3, we conceptualize the interplay of exogenous shocks and BPM in terms of their effects on overall process performance in Sect. 4. Thereafter, we present the identified challenges and opportunities in Sect. 5. We discuss these findings considering extant literature in Sect. 6 and call on the BPM community to address relevant research gaps. We conclude in Sect. 7 with a summary of our work.

## Background

### Exogenous Shocks

Disciplines such as disaster risk science, supply chain management, finance, and economics have already discussed exogenous shocks as well as various related terms. Shi (2019), for example, introduces a framework for studying hazards, disasters, and risks that incorporates a temporal and a process perspective, providing a comprehensive classification of natural or human-induced hazards (i.e., processes or phenomena that may have negative impacts on the economy, society, and ecology) based on their causes and intensity. Disasters, which are direct or indirect consequences of hazards, can lead to crises. Doern et al. (2019) characterize crises as extreme, unexpected, and unpredictable events that create challenges for organizations and require urgent responses. Such crises can be differentiated according to their origins, triggers, scale, and impacts. Based on an extensive overview of the crisis literature, Kuipers and Welsh (2017) distinguish various crisis types (e.g., armed conflict, health, terrorism) and associated themes (e.g., risk, preparedness, decision-making). They conclude that the crisis literature is mainly concerned with natural disasters, preparedness as the predominant theme, and managerial actions to mitigate the negative effects of crises. Björck (2016) consolidates existing crisis typologies based on dimensions such as predictability, controllability, and impact. In line with the concepts just introduced, the term “exogenous shock” is also present in the literature in multiple contexts: primarily economic, political, and financial.

Exogenous shocks have been defined by the International Monetary Fund as “sudden event[s] beyond the control of the authorities that [have] a significant negative impact on the economy” (Geithner 2003, p. 4). They conceptualize crises by emphasizing the external origin of the shock-generating event. A similar concept is that of black swans, referring to highly improbable events with high impact (Taleb 2010). To the best of our knowledge, there is no framework or typology unifying the above-mentioned terms. Rather, the concepts used in the literature reflect subtle nuances concerning the exact nature of relevant events and organizational responses.

Since there is no established understanding of exogenous shocks across disciplines, we define them as *unanticipated, low-likelihood, potentially high-impact events originating from an organization’s environment* (Chakrabarti 2015). Thus, exogenous shocks pose risks (or opportunities) that cannot be fully predicted in advance (Trkman and McCormack 2009). Their occurrence requires substantial organizational reorientation (i.e., simultaneous and discontinuous shifts that transform structures, processes, and control mechanisms (Li and Tallman 2011). Even if organizations account for exogenous shocks, they may de-prioritize them in their risk management strategies, as risks are usually managed based on multiplying their probability with their expected magnitude of impact – and individual exogenous shocks have very low likelihoods (Zsidisin et al. 2004). In the long term, exogenous shocks may force organizations to realign their processes, structures, and strategies to fit the new environment shaped by the exogenous shock.

According to the literature, we posit that exogenous shocks may arise from events of diverse origins (e.g., natural disasters, political crises, healthcare crises, or military conflicts). Importantly, in this research note, we view exogenous shocks from a single organization’s perspective. Thereby, we account for contextual characteristics (Morgeson et al. 2015), meaning that the magnitude and direction of the impact of shock-generating events depend on industry factors and organizational conditions (Li et al. 2017; Li and Tallman 2011). Events that constitute exogenous shocks for one organization or industry may be of marginal or no importance for others. An example is China’s accession to the World Trade Organization in 2001, which specifically led to a significant restructuring of the Portuguese footwear industry and constituted an external shock for related organizations (Corbo et al. 2018). However, the same event may have been of marginal importance for a German restaurant chain.

In terms of impact, events that constitute exogenous shocks according to our definition have been studied in diverse fields (e.g., [macro-]economics, supply chain management, and information systems; (Fedorowicz et al. 2004; Fridgen et al. 2015; Lyytinen and Newman 2008; Singh et al. 2020; Lee 2004). From an economic standpoint, exogenous shocks may entail long- and short-term unfavorable internal conditions, e.g., a loss of human capital causing the unavailability of productive skills or technical knowledge (Noy and Nualsri 2007; Geithner 2003). Some effects of exogenous shocks may be permanent or long-lasting, while others may be temporary. Furthermore, exogenous shocks can substantially impact an organization’s core business or target markets, limiting its access to vital resources or its ability to pursue growth opportunities (Chakrabarti 2015). Most often, an organization’s “old normal” business logic cannot be continued, and a “new normal” must be established (Gersick 1991). Overall, exogenous shocks affect organizations by forcing them to adapt their strategies, business models, structures, and business processes to react to changing conditions and avoid extinction (Martins et al. 2015).

Next to the impact of exogenous shocks, there is ample research on preparing for, responding to, and recovering from unfavorable conditions brought about by exogenous shocks. As the umbrella term, crisis (or disaster) management reflects a continuous process that deals with decision-making, operational activities, actors, and technologies along the three phases of a crisis/disaster (i.e., pre-crisis, crisis, and post-crisis) (Lettieri et al. 2009; Khan et al. 2008; Pearson and Mitroff 1993). Thereby, mitigation and preparedness are the main topics in the pre-crisis stage, disaster response in the crisis phase, and recovery as well as organizational learning in the post-crisis phase (Lettieri et al. 2009; Bundy et al. 2017). In terms of preparedness, organizations strive for resilience, which is a key concept referring to the maintenance of positive adjustments under challenging conditions such that organizations emerge from those conditions strengthened and more resourceful (Vogus and Sutcliffe 2007). Organizational resilience has been extensively studied from multiple perspectives (i.e., capability, process, functional, results) (Chen et al. 2021; Hillmann and Guenther 2021). Examples include investigations on the relationship of resilience to concepts such as flexibility and coping capacity (Karman 2020), defining resilience as an organizational meta-capability (Duchek 2020), and creating conceptual frameworks for the establishment of resilience (Tasic et al. 2020; Kantur and İşeri-Say 2012). As another key concept, high-reliability organizations have emerged as a research stream that examines organizations successfully operating almost error-free even in hostile environments (Roberts 1990; Sutcliffe 2011). In this regard, the idea of collective mindfulness has emerged as heightened alertness to changes/surprises which prioritizes safety over efficiency (Salovaara et al. 2019; Weick et al. 1999). In terms of responding to disasters, business continuity management has been proposed as an approach to identifying, managing, and mitigating risks that may disrupt essential processes and services (Gibb and Buchanan 2006). Moreover, a disaster contingency plan is an essential part of a business continuity plan and includes procedures to perform when disasters occur (Cerullo and Cerullo 2004). Crisis response has also been studied from a human resource viewpoint, with strategic human resource development (HRD) being proposed to enhance the operational capabilities during and capacity to learn after a crisis (Wang et al. 2009). In the post-crisis phase, organizational learning has been investigated in terms of organizations’ ability to derive insights from tackling disasters/crises (Broekema et al. 2017). Thereby, promoting organizational learning also prior to and during crises has been shown to generate favorable effects in all stages of crisis management (Wang 2008).

While the mentioned approaches do not fully cover the multi-faceted nature of research on crisis management and related approaches, they provide an overview of important research streams related to organizations’ preparedness, response, and recovery from crises/disasters. Thus, they are also highly relevant in the context of exogenous shocks as a specific form of crisis. We revisit the topics listed above in Sect. 6 when discussing the implications of our results for BPM and related research areas.

### Business Process Management and Process Change

BPM is the science and practice of overseeing how work is performed to ensure consistent outcomes and capitalize on improvement opportunities (Dumas et al. 2018; van der Aalst 2013). It “consolidates how to best manage the (re-)design of individual business processes and how to develop a foundational capability in organizations catering for a variety of purposes and contexts” (vom Brocke and Rosemann 2015, p. viii). BPM is commonly structured through capability frameworks that include capability areas conducive to establishing process orientation in organizations (Poeppelbuss et al. 2015; Rosemann and vom Brocke 2015; Van Looy 2020). One of the most widely adopted BPM capability frameworks is that of de Bruin and Rosemann (2007), which groups capability areas according to six core elements of BPM – strategic alignment, governance, methods, information technology (IT), people, and culture – that have been extensively used in BPM research (Van Looy et al. 2017; Kerpedzhiev et al. 2020; vom Brocke and Mendling 2018). Table 1 provides brief definitions of these core elements, which we use to structure the challenges and opportunities of exogenous shocks for BPM.

**Table 1:** Definitions of the six core elements of BPM (de Bruin and Rosemann 2007)

Core element	Definition
Strategic alignment	The continual tight linkage of organizational priorities and enterprise processes enabling achievement of business goals
Governance	Establishing relevant and transparent accountability and decision-making processes to align rewards and guide actions
Methods	The approaches and techniques that support and enable consistent process actions and outcomes
Information technology	The software, hardware, and information management systems that enable and support process activities
People	The individuals and groups who continually enhance and apply their process-related expertise and knowledge
Culture	The collective values and beliefs that shape process-related attitudes and behaviors

In the BPM context, exogenous shocks represent a specific form of process change. According to the BPM literature, various types of process change can be distinguished based on dimensions such as intentionality, frequency, scope, degree of change, duration, or performance effects (Grisold et al. 2019; König et al. 2018). To differentiate exogenous shocks from other types of process change and to specify its effects on processes, we focus on the *intentionality* and *degree of change* dimensions, perceiving process change as either intentional or unintentional and as incremental or radical. Table 2 combines both dimensions and lists examples, which have been studied to a varying extent in the literature.

**Table 2:** Exemplary types of process change

	Intentional	Unintentional
Incremental	Continuous process improvement	Process drift
Radical	Process reengineering, process innovation	Exogenous shock, process disruption

Beginning with intentional change, business process reengineering is an example of radical change (Hammer and Champy 2006), whereas continuous process improvement (e.g., lean management) implies incremental change (Davenport 1997). The distinction between radical and incremental process change is also considered in the literature on ambidextrous BPM in terms of process exploration (opportunity-driven, radical change) and exploitation (problem-driven, incremental change) (Rosemann 2014; Grisold et al. 2019). Process drift, which is related to the gradual change of processes (van der Aalst et al. 2012; Pentland et al. 2020; Beverungen 2014), is an example of unintentional, incremental process change.

In contrast to the other types of process change, unintentional radical process change, which could result from an exogenous shock, has received little attention in BPM research. However, two important related concepts established in BPM research are process resilience and agility (Chen et al. 2014; Antunes and Mourão 2011; Rosemann 2020; Gilbert et al. 2012). Process resilience relates to flexibility-by-design (i.e., the ability to incorporate alternative execution paths and fast change ad-hoc at design time; Schonenberg et al. 2008) and, thus, facilitates the organizations’ preparedness to address exogenous shocks. In contrast, process agility is associated with flexibility-by-deviation, allowing organizations to quickly react to environmental changes by deviating from prescribed process execution paths at runtime. Thus, both concepts directly relate to the pre-shock and in-shock phase and are cognate with the concepts mentioned in Sect. 2.1. Further, BPM research has also addressed organizational learning and knowledge management (Shelagowski 2015; Jung et al. 2007; Choi et al. 2004). Despite their potential to support and enrich the knowledge base regarding the handling of exogenous shocks throughout all phases, the mentioned concepts from the BPM literature have not been investigated in relation to exogenous shocks yet. Therefore, we set out to explore the intersection of BPM and exogenous shocks as a form of radical, unintentional process change against the background of relevant concepts from related disciplines. While we do not claim exhaustiveness, Table 3 provides a simplified overview of the discussed BPM concepts and those from Sect. 2.1 regarding their primary relevance prior to, during, and in the aftermath of exogenous shocks. Thereby, an unambiguous mapping of the concepts is unfeasible since they cover a wide spectrum of ideas, which normally has implications for all phases.

**Table 3:** BPM and further concepts related to tackling exogenous shocks

	Pre-shock phase	In-shock phase	Post-shock phase
BPM research	Process resilience; flexibility-by-design	Process agility; flexibility-by-deviation	–
Related research	Organizational resilience	Business continuity; disaster recovery	Organizational learning

## Research Approach

**Fig. 1:**
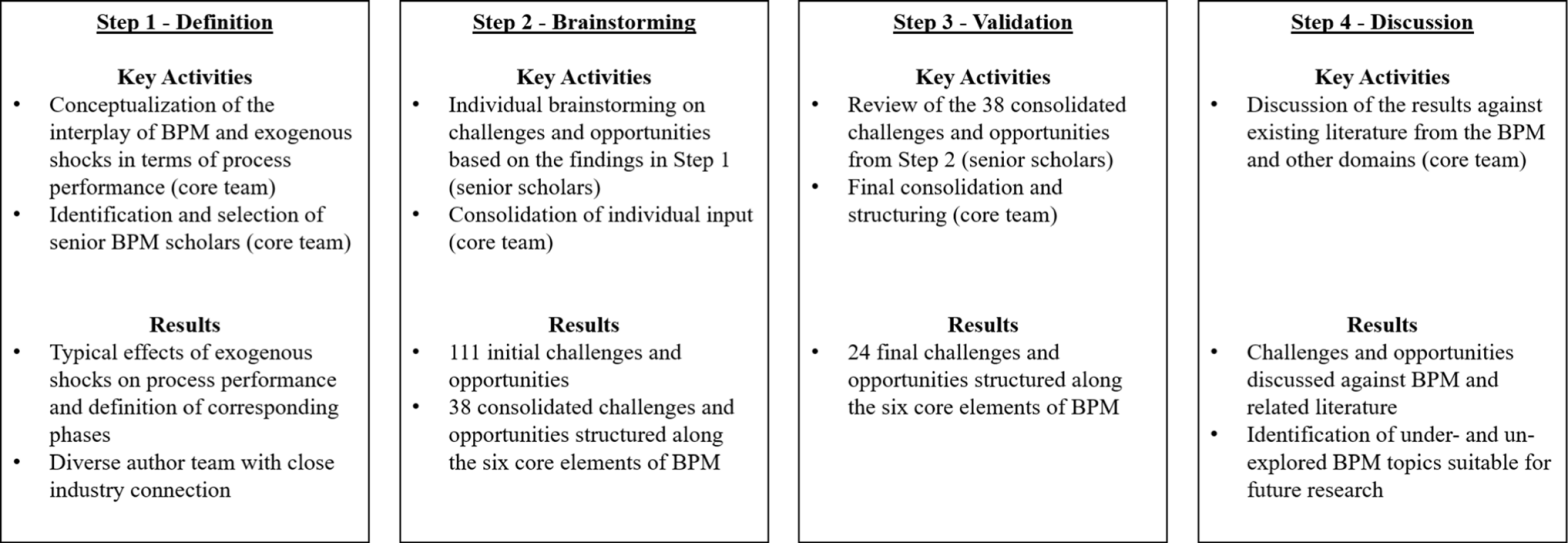
Overview of the research approach

To answer our research question, we followed a four-step research approach informed by the blueprint of ranking-type Delphi studies (Dalkey and Helmer 1963; Paré et al. 2013): *definition*, *brainstorming*, *validation*, and *discussion.* Figure 1 provides an overview of the research approach.

In the *definition* step, the initiating four co-authors of this paper (core team) conceptualized the interplay of BPM and exogenous shocks (Sect. 4). This conceptualization served as a foundation for structuring the subsequent steps. The core team then invited senior BPM scholars, all of whom have close connections with industry, to contribute to the identification of challenges and opportunities for BPM in the context of exogenous shocks and asked them to join the author team. Following Olbrich et al. (2015) as well as established expert selection criteria for Delphi studies (Okoli and Pawlowski 2004), we ensured diversity in terms of geographical activity, gender, and technology/management focus. With this, we aimed at achieving as comprehensive a perspective of the impact of exogenous shocks as possible.

In the *brainstorming* step, the author team engaged in several rounds of idea generation and consolidation. Based on an initial exchange, all scholars individually brainstormed challenges and opportunities for BPM due to exogenous shocks as is typical in the related phases of Delphi studies (Paré et al. 2013). Thereby, they drew on their observations of how organizations had reacted to exogenous shocks in the past. To structure their input and the overall results, we used the six core elements of BPM introduced in Sect. 2 and the phase model presented in Sect. 4. The brainstorming resulted in an initial list of 111 challenges and opportunities. The core team consolidated the input following a consensus-oriented interpretivist paradigm promoted by the diversity of viewpoints on BPM. This approach follows an established epistemic theory of truth (Becker and Niehaves 2007). The core team members read the full lists and clustered the input using open coding (Glaser 1978; Strauss and Corbin 1991). To offset potential bias, the core team paid particular attention to avoiding challenges and opportunities that only related to the COVID-19 pandemic, which was omnipresent when this research was conducted. This procedure was repeated two times until consensus was reached (Butler 1998). This resulted in 38 preliminary challenges and opportunities.

In the *validation* step, all co-authors reviewed the consolidated list to ensure that their individual input had been appropriately incorporated and that the identified challenges and opportunities were consistent in terms of content and concept. They also provided feedback on the consolidated list, which was then incorporated by the core team. This step resulted in 24 challenges and opportunities for BPM, which are presented in Sect. 5. Since the author team agreed to include only those two challenges and two opportunities per core element of BPM in the results that are reflected the most in the input of all co-authors, we do not claim that our results are exhaustive. Rather, we see them as “food for thought” and stimuli for future research.

Finally, in the *discussion* step, the identified challenges and opportunities were discussed against existing literature from BPM and other domains, predominantly getting back to concepts from Sect. 2. The idea was to identify relationships, under- and un-explored topics as well as potential avenues for future research. The results of the discussion are presented in Sect. 6.

## Interplay of BPM and Exogenous Shocks

While intentional process change typically leads to positive performance effects, exogenous shocks commonly have adverse effects on process performance, which in turn is an important driver of organizational performance (Hammer 2015; Lehnert et al. 2016). As pointed out in Sect. 2.1, the magnitude of such effects depends on the nature of the shock-generating event as well as on industrial and organizational conditions. Hence, the impact of exogenous shocks needs to be contextualized (vom Brocke et al. 2015).

**Fig. 2:**
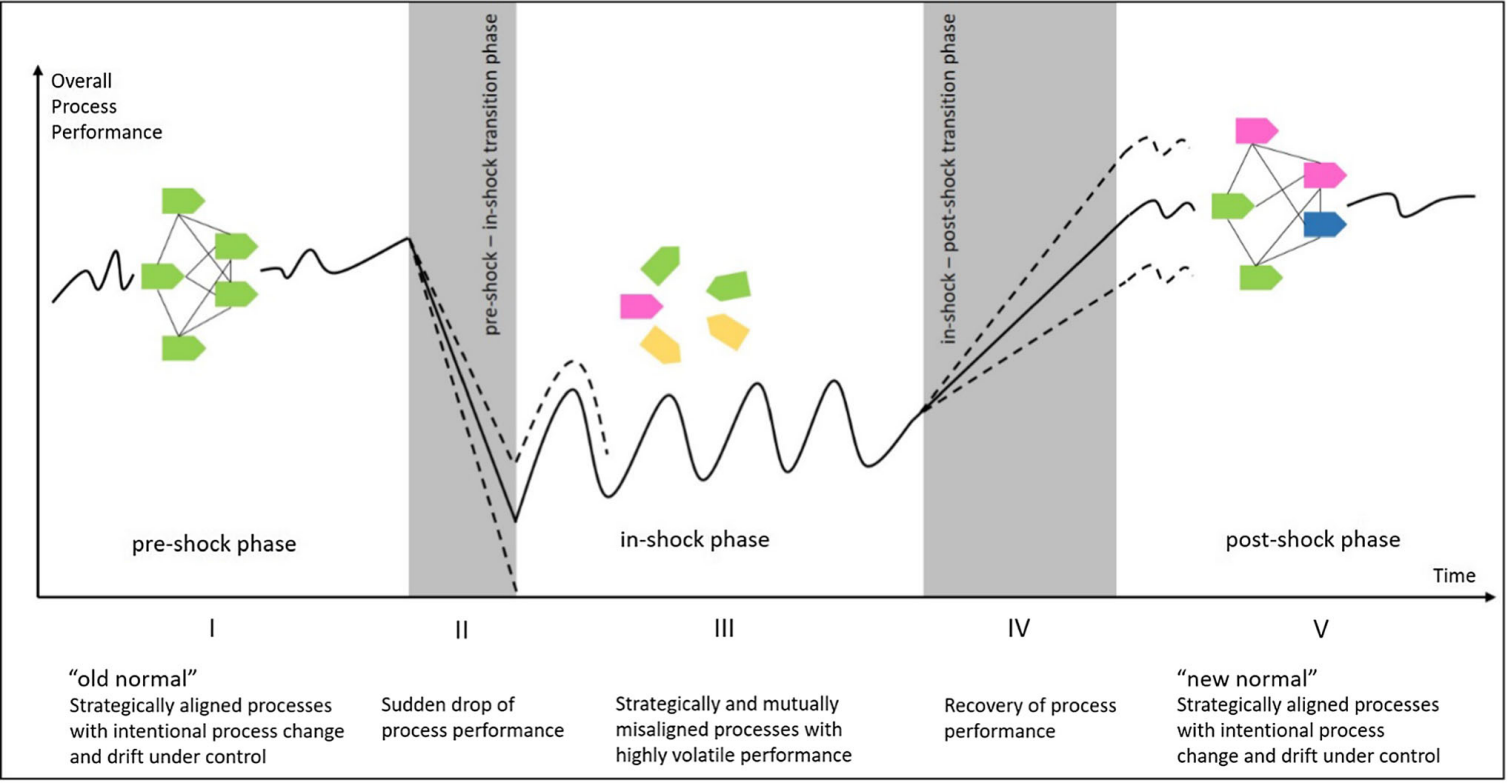
Potential effects of exogenous shocks on organizations’ overall process performance

Due to their unexpected nature and adverse effects, exogenous shocks are notorious for their potential to disrupt business processes and their possibly existence-threatening consequences for organizations. In the following, we conceptualize the effects of exogenous shocks on organizations’ overall process performance. We cross-referenced these effects with extant literature on disaster and crisis management and related phases (Sect. 2.1). Transferred to the context of exogenous shocks, the pre-shock phase usually involves preparatory activities, the in-shock phase encompasses the direct impact of and response to exogenous shocks, and the post-shock phase comprises recovery activities. Figure 2 shows the potential courses of an organization’s overall process performance in response to an exogenous shock, ranging from one steady state (with limited volatility and possibly drift under control) before the occurrence of an external shock (“old normal”) to another steady state (with limited volatility and maybe a positive or negative controlled drift) after the organization has adapted to the post-shock environment (“new normal”). In line with the literature on crisis management and discussions within the author team, we distinguish five phases (I to V) with distinct effects on process performance, which we present below.

Exogenous shocks impact business processes in two ways: First, they occur unexpectedly and disrupt organizations’ external environments, thereby creating also internal uncertainty that renders the pre-shock (I) business processes and ongoing change initiatives (e.g., process improvement roadmaps) inadequate. Hence, exogenous shocks impact overall process performance negatively in the pre-shock-in-shock transition phase (II) and may even drive organizations out of existence. In some cases, exogenous shocks can have a positive demand-side effect for some organizations (i.e., as for video conferencing providers during the COVID-19 pandemic). Nevertheless, overall process performance is still likely to suffer due to limited capacity or longer lead times. Second, as organizations begin to reconfigure their business processes to respond to the conditions caused by the shock, they experience highly volatile process performance due to great uncertainty and the internal disordering of processes in the in-shock phase (III). In this phase, exogenous shocks can create “aftershocks,” cycling between the two aforementioned effects (e.g., the following waves of the COVID-19 pandemic). However, with decreasing volatility and increasing adaptation to the new environment, organizations regain overall process performance as they transition from the in-shock to a post-shock phase (IV). This transition needs not to be continuous. Rather, organizations may “leapfrog” to the post-shock phase (V), if they substantially change their processes or successfully launch process, business model, or product innovations. An organization’s process performance in the post-shock phase (V) may be lower than, equal to, or higher than that in the pre-shock phase, depending on contextual and organization-specific factors (e.g., the availability of a recovery stimulus package, the capability to adapt business models to new external conditions, or the market entry of new competitors during the shock). Notably, phases I to V can vary in length according to the nature of the exogenous shock and the organization’s specific context.

BPM can contribute to mitigating the adverse effects of exogenous shocks in multiple ways. First, it can help reduce the initial drop in process performance in the pre-shock–in-shock-transition phase (II). Second, it can reduce the volatility of process performance in the in-shock phase and shorten the duration of both the in-shock phase (III) and the in-shock-post-shock transition phase (IV). Finally, BPM can help ensure that process performance in the post-shock phase (V) stabilizes at a higher level than that of the pre-shock phase (“bounce forward”) and to prevent stabilization at a lower level (“bounce back”). Thus, BPM can simultaneously contribute and benefit from approaches related to organizations’ preparedness (e.g., resilience), response (e.g., business continuity, disaster recovery), and dealing with the aftermath of exogenous shocks (e.g., organizational learning) to mitigate and possibly capitalize on the effects of exogenous shocks. We refer to the phases just introduced when elaborating on the challenges and opportunities in the following Sect. 5.

## Challenges and Opportunities for BPM

In line with our research approach, we compiled challenges and opportunities of exogenous shocks for BPM (Table 4), which we structure and present following the six core elements of BPM. When presenting the challenges and opportunities, we also refer to the phases introduced in Sect. 4. We added examples from diverse contexts to make the challenges and opportunities tangible.

**Table 4:** Challenges and opportunities of exogenous shocks for BPM

Core element	Challenges	Opportunities
Strategic Alignment	C1. Sudden obsolescence of organizational strategy and uncertainty regarding the permanence of changes C2. Enforced reprioritization of business process improvement efforts	O1. Need for novel (potentially process-based) value propositions and radical improvement of existing business processes O2. Improvement of process-enabled shock resilience
Governance	C3. Sudden inadequacy of existing BPM and process governance setups C4. Need for fast switches between different governance modes	O3. Development of robust, multi-context BPM and process governance O4. Potential to instill process-oriented governance in an organization’s “DNA”
Methods	C5. Lack of agile process (re)design methods C6. Inadequacy of existing process roll-out and change management methods	O5. Development of simplified and resilient business processes O6. Insights into the vulnerability of business processes
Information Technology	C7. Absence of scalable and remotely available process management tools C8. Obsolescence of existing process monitoring setups	O7. Adoption of lightweight process automation, deployment, and experimentation techniques O8. Increased transparency through increased digitalization
People	C9. Absence of scalable process training concepts C10. High individual stress owing to misaligned business processes, reset of experience curve effects, and communication overload	O9. Scaling of organization-wide process thinking and digital literacy O10. Leveraging the creative potential of employees for process improvement
Culture	C11. Potential deprioritization of customer orientation at the expense of internal shock management C12. Necessity to effectively unlearn existing business processes	O11. Utilization of the shock experience as a foundation for future radical process changes O12. Transition toward a results-oriented culture of trust with improved work–life balance

### Strategic Alignment

Owing to exogenous shocks, the alignment between business processes and BPM capability, on the one hand, and organizational strategy, on the other, becomes strained. Exogenous shocks can render existing strategies obsolete and enforce an adaptation of business models and processes; for instance, the COVID-19 pandemic has caused educational institutions to quickly adopt online platforms and come up with new educational offerings (Seetharaman 2020). Organizations have different priorities regarding their objectives in the pre-shock (I), in-shock (III), and post-shock (V) phases, but existing processes typically no longer meet the right business objectives. Moreover, since exogenous shocks can render organizational strategy obsolete, both process and BPM objectives must be scrutinized. Building on these observations, we propose two challenges and opportunities for BPM.

- *C1: Sudden obsolescence of organizational strategy and uncertainty regarding the permanence of changes.* BPM needs to support the fast switch between pre-shock (I), in-shock (III), and post-shock (V) strategies. Following the arguments in Sect. 4, BPM can help in identifying those process and strategic elements that can stay as-is, mitigating the negative effects on process performance. Hence, BPM needs to support dynamic strategic realignment, since there may be no consistent in-shock strategy and contexts may change rapidly within and outside organizations. Moreover, in the in-shock phase (III), BPM needs to support the identification of permanent and temporary changes to allow organizations to appropriately respond to the shock on the operational and strategic levels.
- *C2: Enforced reprioritization of business process improvement efforts.* In the event of an exogenous shock, organizations may need to cancel or postpone greenlighted process improvement initiatives in the in-shock phase (III). BPM should therefore equip process managers with instruments to differentiate must-do projects from those that have been rendered obsolete or have lost their strategic fit. Moreover, it is vital to identify projects that should be initiated or continued even in the in-shock phase (III).
- *O1: Need for novel (potentially process-based) value propositions and radical improvement of existing business processes.* BPM should not only help to operationalize organizational strategies, but also actively shape them. BPM professionals can use their process and domain knowledge to design novel process-based value propositions in both the in-shock (III) and post-shock (V) phases. Based on this foundation, they can design strategies that improve the strategic alignment of business processes and BPM and support the organization in tapping into new revenue pools.
- *O2: Improvement of process-enabled shock resilience.* BPM should drive organizational resilience. Resilient organizations depend on resilient business processes (i.e., processes designed with alternative execution paths or sufficient degrees of freedom for dynamic adaptation at runtime). Such organizations have low latency in response to shocks since processes either do not need to be redesigned at all or only require partial redesign.

### Governance

After an exogenous shock (i.e., beginning with the pre-shock–in-shock transition [II]), existing decision-making processes and authorities are confronted with highly uncertain and volatile environmental conditions, which nevertheless require fast and concerted action. An extreme example is the Fukushima nuclear disaster in 2011, which required urgent decisions with enormous consequences in a highly uncertain environment (Travadel 2017). In such situations, established roles change due to the need for rapid process adaption, and process performance indicators lose their relevance or become ineffective in the in-shock phase (III). This poses challenges and opportunities for BPM practitioners.

- *C3: Sudden inadequacy of existing BPM and process governance setups.* Established and commonly applied governance mechanisms are not practical during the pre-shock–in-shock transition (II) and in-shock (III) phases. This is for two reasons: First, the focus of interest and related business objectives change in the event of exogenous shocks. Second, data collection for key performance indicators becomes more challenging, especially when underlying information systems are not process-aware. Moreover, many routine processes become ineffective, making it challenging to ensure compliance because reference points in terms of to-be processes are no longer available.
- *C4: Need for fast switches between different governance modes.* The use of a proprietary governance mode in the in-shock phase (III) can contribute to maintaining the pre-shock level or at least mitigating decreases in process performance. In the sense of process continuity, organizations need plans for the temporary and permanent simplification of governance processes. Such simplification includes roles, responsibilities, and methods that replace existing approaches in the in-shock phase (III). One example is the International Red Cross/Red Crescent movement, which, in the case of a disaster, switches governance structures to a crisis mode to establish working processes where relief is needed (Peterken and Bandara 2015). The challenge here is to incorporate the possibility of a targeted switch back to the “old normal” state (if the expected shock does not materialize or is not long-lasting) or the transition to an improved “new normal” state (IV/V).
- *O3: Development of robust, multi-context BPM and process governance.* In the event of exogenous shocks, intentional process change increasingly takes place in ad hoc and bottom-up initiatives, which need to be managed through lightweight and adaptive governance setups. As discussed, the need for such setups is magnified by exogenous shocks because the increased speed and extent of process change can lead to misinformation and confusion. By contextualizing and synchronizing decentral ad-hoc changes, BPM can implement both robust and multi-context BPM and process governance.
- *O4: Potential to instill process-oriented governance in an organization’s “DNA.”* The implementation of new governance setups may facilitate transformation toward a truly process-oriented organization. Currently, many organizations feature BPM roles and responsibilities that are formally described but not anchored in the organizations (e.g., process owners without any decision-making rights or budgets). Exogenous shocks can help BPM practitioners to implement truly process-oriented governance structures.

### Methods

Organizations do not normally rely on dedicated methods when reacting to exogenous shocks. Rather, they try to find ad-hoc solutions for upcoming problems. This was partially the case in several of the major space shuttle disasters NASA faced, in the wake of which the agency embraced some new safety procedures (Donahue and O'Leary 2012). However, ad-hoc solutions are not the product of properly managed projects but of taskforces solely focused on an organization’s survival (e.g., aimed at reducing the initial drop in performance brought about by the shock [II]). Thus, exogenous shocks present challenges and opportunities for organizations from a BPM methods perspective.

- *C5: Lack of agile process (re)design methods.* Exogenous shocks call for agile process (re)design methods. Organizations that use such methods can reduce initial performance decreases and recover more quickly (II/IV). This is true because BPM typically works in a deductive way – from symptoms (i.e., process inefficiencies) to solutions (i.e., optimized processes) based on existing frameworks (e.g., process improvement patterns). Since inductive process optimization (e.g., based on process mining) does not work well in the event of exogenous shocks, abductive process (re)design methods are needed. Such methods contribute to better process design based on the changing environment without relying on existing frameworks (deduction) or vast data sets (induction).
- *C6: Inadequacy of existing process roll-out and change management methods.* It is not only crucial for organizations to identify viable process configurations for the in-shock (III) and post-shock (V) phases, but also to implement them. Standard processes originally stemming from the pre-shock phase (I) may additionally need to be split into multiple process variants. Thus, methods such as the rapid prototyping of several process variants in combination with strong change management capabilities are needed to speed up the recovery (IV) from exogenous shocks.
- *O5: Development of simplified and resilient business processes.* Organizations are rarely prepared to face exogenous shocks through shock-resistant and possibly simple process designs. While some organizations may not need to change processes that are flexible enough, for others, exogenous shocks present an opportunity to simplify historically developed processes and, where possible, improve process resilience as well as process performance in the post-shock phase (V).
- *O6: Insights into the vulnerability of business processes.* Especially organizations with resilient processes already have deep insights into related process vulnerabilities. They can easily switch from a “normal” mode to a “shock” mode with minimal impact on in-shock (III) process performance. Organizations that rely on agile process change benefit from prior insights into vulnerabilities, since they allow for a rapid focus on critical processes during the in-shock phase (III).

### Information Technology

Information technology (IT) that supports process execution can contribute to or impede process agility and resilience; for example, GeoWeb technologies have been used by various organizations to help deploy emergency-related Web applications for visualizing the impact of natural disasters such as hurricanes and widespread fires (Roche et al. 2013). Process change implies changes in information systems, which can be achieved through process-aware technology or large-scale IT change. BPM-related information systems commonly used to support the design, modeling, or monitoring are only partially useful in the in-shock phase (III); therefore, BPM is facing the following challenges and opportunities.

- *C7: Absence of scalable and remotely available process management tools.* Regarding process design and modeling, tools can only be used if they are widely (in the case of COVID-19, remotely) available. For instance, it is possible that organizations lacking cloud solutions with scalable license models will be unable to make use of their design tools when adapting to a “new normal” (VI). Such tools can be used in the in-shock phase (III) only if knowledge about them is broadly available.
- *C8: Obsolescence of existing process monitoring setups.* Information systems for monitoring and controlling business processes need to be adapted quickly. Control mechanisms that rely on experience become worthless if the experience does not match the new in-shock/post-shock reality (III/V); hence, organizations need to quickly adapt their process monitoring tools.
- *O7: Adoption of lightweight process automation, deployment, and experimentation techniques.* Hard-wired business processes in heavyweight IT are challenged by the rapid changes induced by exogenous shocks. Organizations that rely on adaptive process-aware information systems can experiment with new process designs and deploy them quickly into production. Moreover, lightweight solutions, such as Robotic Process Automation or pre-configured chatbots, can enable the fast scaling of new processes and, hence, help in coping with shocks (III/VI).
- *O8: Increased transparency through increased digitalization.* Especially regarding the COVID-19 pandemic, which required substantial remote work and customer interaction, there was an increased effort to digitalize transactions between users and employees. Organizations can leverage these advances to accelerate process digitalization; for example, by using data-driven process technology to quickly improve in-shock processes and achieve or even surpass pre-shock performance (IV/V).

### People

Exogenous shocks are not only a challenge to organizations at large but also to process participants (including process managers); for instance, human resources managers of companies affected by the attacks of September 11, 2001, had to make potentially existence-threatening decisions regarding moving work locations and/or hiring additional staff to ensure stable operations (Sayegh et al. 2004). In such cases, people need to rapidly find and sustain new ways of working, which places employees under pressure, since training for and during exogenous shocks is often not possible. Thus, exogenous shocks present BPM with challenges and opportunities from a people-oriented perspective.

- *C9: Absence of scalable process training concepts.* In many organizations, BPM skills tend to be centralized (e.g., in process centers of excellence), but to cope with shocks, BPM skills need to be distributed across organizations and process change needs to be empowered (Kaplan et al. 2020). Moreover, the implementation of new processes requires employees to acquire new skills and adapt to changing roles. Accordingly, providing continuous and comprehensive process guidance that enables process participants to quickly adapt to new or changed processes in the in-shock and post-shock (III/V) phases is challenging.
- *C10: High individual stress owing to misaligned business processes, reset of experience curve effects, and communication overload.* Especially in an environment of decentralized process change, interfaces between processes may be misaligned; hence, leadership needs to reduce individuals’ job strain when processes do not work seamlessly in the in-shock and post-shock (III/V) phases. Particularly when transitioning to the post-shock phase (IV), organizations should employ change management initiatives to ensure that people do not revert to old habits. Only then can organizations reach higher levels of performance after the shock than before the shock (V).
- *O9: Scaling of organization-wide process thinking and digital literacy.* BPM practitioners have an opportunity to increase the digital literacy of employees due to the wider adoption of digital technologies and process-aware information systems. This unprecedented openness toward (emerging) digital technologies and the acquired literacy can catalyze further process digitalization after a shock (V).
- *O10: Leveraging the creative potential of employees for process improvement.* In response to exogenous shocks, organizations have a unique opportunity to harvest the creative potential of employees for improving their business processes. Employees’ efforts to “make things work” in the in-shock phase (III) and the corresponding potential for positive deviance can be disseminated within organizations.

### Culture

Organizational and BPM culture becomes strained by an exogenous shock. For instance, the fundamental shift in the smartphone market due to the introduction of the iPhone in 2007 caused widespread fear and a disconnect between top and middle management at Nokia (Vuori and Huy 2016). A culture of high commitment to existing but obsolete objectives may cause problems if an exogenous shock occurs, while commitment to measures for coping with the shock is important; hence, BPM practitioners face challenges and opportunities regarding BPM culture.

- *C11: Potential deprioritization of customer orientation at the expense of internal shock management.* When organizations focus too greatly on securing their own survival in the in-shock phase (III), interactions with partners and especially customers may suffer. In this regard, a BPM culture dedicated to customer orientation is highly desirable. Organizations need to ensure that the deprioritization of customer orientation – if needed at all – is a temporary and conscious decision.
- *C12: Necessity to effectively unlearn existing business processes.* Process change can benefit from a healthy level of process commitment and the corresponding ability to unlearn past routines during a shock (III). However, process commitment should not focus only on as-is processes, but also on achieving overall process goals. In times of exogenous shocks (II/III), an overcommitment to, and reliance on, existing processes may prove to be a liability for organizations by causing a sharp decrease in process performance and, potentially, leading to the demise of the organizations.
- *O11: Utilization of the shock experience as a foundation for future radical process changes.* From a cultural perspective, an exogenous shock might be a good “burning platform” for future radical process change. Based on previously experienced exogenous shocks, process managers can refer to changes made because of the shock whenever the feasibility of future process changes is challenged in the post-shock phase (V).
- *O12: Transition toward a results-oriented culture of trust with improved work–life balance.* During the COVID-19 pandemic, organizations had an opportunity to change from an attendance-oriented culture toward a results-oriented culture. While such transitions pose challenges for both line and process managers in leading people, they also provide opportunities to explore new and hopefully better ways of working with an improved work–life balance (e.g., working from home).

## Discussion

We now discuss the identified challenges and opportunities against the literature from BPM and related domains. Thereby, we point to opportunities for advancing both BPM research and research related to the management of exogenous shocks as well as their symbiotic relationship. We specifically get back to corresponding concepts introduced in Sect. 2. Just like the challenges and opportunities, this section is structured according to the six core elements of BPM.

### Strategic Alignment

As for the core element strategic alignment, BPM has the potential to complement organizational resilience through a process-based approach (O2). Some research initiatives already aim at investigating the connection between resilience and information systems in general (Müller et al. 2013) with the intersection of BPM and resilience also being explored (Zahoransky et al. 2015; Antunes and Mourão 2011). BPM research should build on such works to establish guidance regarding the identification and assessment of critical processes in line with corporate strategy, which should be resilient to sustain essential organizational functions in the event of exogenous shocks. In this regard, examining established approaches related to process prioritization can be very valuable in determining processes’ criticality both individually and based on their interconnectedness (Lehnert et al. 2018; Kreuzer et al. 2020).

In the wake of exogenous shocks, established business strategies and processes can become obsolete (C1). Hence, organizations may need to re-evaluate the maturity of their BPM capabilities. Building upon prior publications (e.g., Poeppelbuss et al. 2015), BPM research should guide organizations in developing capabilities to achieve a new BPM/strategy fit. Moreover, organizations experience dysfunctional processes and must balance between a swift recovery of core processes and the development of long-term process improvement strategies to address the medium- and long-term impact of shocks. The magnitude of shock effects across all phases is an important context factor for prioritizing process improvement initiatives (C2). Therefore, research on process performance metrics should be geared toward exogenous shocks and reflect strategic priorities (Van Looy and Shafagatova 2016; Estrada-Torres et al. 2019). Moreover, BPM research should help organizations explore value propositions that fit the “new normal” (O1) in the post-shock phase. Related approaches may build on existing process-led value propositions (e.g., Johannsen 2018) as well as on findings from explorative BPM to guide the derivation of new value propositions (Grisold et al. 2021).

### Governance

Our results have several implications from a governance perspective. To begin with, exogenous shocks require switching between context-sensitive governance models that consider external and internal factors (C4, O3). As outlined in Sect. 2, such an approach requires an understanding of different types of exogenous shocks. For example, while shocks such as the Fukushima nuclear disaster had a major impact, the duration and nature of that shock differ greatly from that of the COVID-19 pandemic. In this regard, BPM research can build upon existing typologies of crises, e.g., from Kuipers and Welsh (2017), to distill shock archetypes requiring similar BPM approaches. Moreover, the phase model from Fig. 2 is also context-sensitive, meaning that the magnitude and duration of effects depend on internal and external factors (e.g., an organization already operating in a highly uncertain environment may respond better to shocks). In this regard, context-aware BPM has been recognized as an important topic area and extensively studied (vom Brocke et al. 2015; vom Brocke et al. 2021a; Santoro et al. 2017). BPM research should extend this idea to identify contextual factors relevant for exogenous shocks and investigate their influence on BPM and process governance (Kerpedzhiev et al. 2020). By identifying context-sensitive BPM governance models depending on shock archetypes, BPM can also contribute to extending and operationalizing crisis response according to established disaster taxonomies and typologies (Kuipers and Welsh 2017; Björck 2016).

In terms of the need for new governance setups in the context of exogenous shocks (C3), understanding the relationship between process flexibility and organizational resilience is key. Even though flexibility and resilience are not identical concepts, current research on resilience shows that both concepts are positively linked (Duchek 2020; Karman 2020). In this regard, BPM must clarify the role of workarounds and process deviance, which have received significant attention in recent BPM research (König et al. 2018; Alter 2014; Beerepoot et al. 2019; Weinzierl et al. 2021). One of the identified opportunities relevant to BPM governance is the occurrence of positive deviance, which should be identified and scaled (O10). However, without clear guidance deviance entails risks. Thus, BPM research should develop frameworks that examine appropriate conditions, processes, and levels of deviance in the context of exogenous shocks.

### Methods

The challenges and opportunities related to the core element methods imply that BPM should extend its methodological base to appropriately cover exogenous shocks. As mentioned, the priority in dealing with exogenous shocks is given to the immediate organizational response and maintaining business continuity. Business continuity research has not only recognized the need to enhance the preparedness for incidents but also to support organizations in responding to them (Niemimaa 2015). Translated to the BPM context, organizations need to take quick actions in the in-shock phase to address nonfunctional processes and critical process performance. This entails the need for agile BPM approaches, methods, and systems that allow for bottom-up design, implementation, and rollout of new processes (C5) as well as to support change management initiatives (C6). Thus, existing research on agile BPM methods should be extended to the use case of short-term handling of exogenous shocks (Thiemich and Puhlmann 2013). In addition, BPM must support organizations in resource allocation and decision-making regarding balancing short-term emergency handling and far-reaching, strategic process change (C2). Thereby, established ideas in BPM research such as process project portfolio management as a means to balance process improvement and BPM capability development (Lehnert et al. 2017) can be adopted to the case of exogenous shocks.

As for the design of resilient processes (O5), actionable process-based mechanisms that enable process resilience are needed (e.g., Antunes 2010). Such mechanisms could build upon existing works regarding process flexibility and enable integrating redundant/alternative process paths for critical processes. Thereby, as mentioned in Sect. 2, flexibility-by-design and flexibility-by-deviation are of interest in enhancing the resilience of business processes at design- and run-time (Schonenberg et al. 2008). By using business processes as the unit of analysis, BPM can contribute to instrumentalizing organizational resilience – a challenge, which has been recognized in the corresponding research stream (Annarelli and Nonino 2016).

Finally, existing approaches in measuring resilience (Chen et al. 2021) can be strengthened through a BPM lens to achieve real-time process resilience monitoring and an appropriate ex-post evaluation (O6). The latter could be supported by process mining methods, which deliver insights into process vulnerabilities based on event log data (Koslowski et al. 2013).

### Information Technology

Our results also have implications on IT-related BPM topics. In conjunction with the opportunity regarding process automation (O7), BPM research should address the potential of digital technologies for dealing with sudden and unexpected events. Some research initiatives initiated in the wake of the COVID-19 pandemic show promising results regarding the potential of digital technologies in enhancing organizational resilience (Syed et al. 2020; Marques da Rosa et al. 2021; Marcucci et al. 2021; Kregel et al. 2021). In the context of BPM, digital technologies can help make processes more responsive and potentially more predictive regarding changing external conditions. Therefore, BPM research should build on existing initiatives regarding the exploration of the opportunities offered by digital technologies (Denner et al. 2018), specifically in enhancing real-time monitoring and prediction of disruptions as well as process transparency (C8, O8) (e.g., Ivanov and Dolgui 2021).

Further, rapid process change resulting from exogenous shocks requires flexible IT architectures (C7). In this regard, process-aware information systems have already been researched with a focus on flexibility and the handling of declarative process models (Reichert and Weber 2012; Di Ciccio et al. 2017). Research on such systems could be informed by works on high reliability organizations, which address the “frame problem” concerning the inability of algorithms to adapt to conditions outside their developers’ cognitive frame (Salovaara et al. 2019) and in general have the ability to reduce process agility (Plattfaut and Borghoff 2022). At the same time, exploring the development and validation of process-aware information systems considering existing frameworks for resilient BPM (e.g., Antunes and Mourão 2011) can help better understand the role of IT in crisis management (Sakurai and Murayama 2019).

### People

From a people perspective, BPM research should first evaluate the possibility of transferring skill portfolios as well as communication and training models (C9) from the human resources and emergency management domains. Second, BPM can help structure and sustain organizational learning efforts. In this regard, works on organizational learning in the context of crises (e.g., Antonacopoulou and Sheaffer 2014) can be combined with approaches at the intersection of knowledge management and BPM (e.g., Jung et al. 2007; Choi et al. 2004) to provide guidance for fast organizational learning and knowledge dissemination in the case of exogenous shocks. Thereby, BPM also has the potential to provide a systematic approach to learning and ensure that shock-related knowledge including newly acquired competencies (O9) remain available in the post-shock phase.

Apart from the implications on training and knowledge management, facing non-functional processes may put employees under pressure (C10). In this respect, the pivotal role of HRD has been recognized in guiding organizations through crises (Wang et al. 2009). Therefore, BPM research should evaluate how to adapt results from HRD, such as leadership behavioral patterns and styles, thus contributing to shaping requirements for process leaders in times of exogenous shocks (Dirani et al. 2020; Bowers et al. 2017).

### Culture

In terms of culture, BPM and crisis management research alike should examine to what extent established BPM cultural values (i.e., customer orientation, excellence, responsibility, and teamwork (Schmiedel et al. 2015)) are neglected, strengthened, or substituted in the event of exogenous shocks (C11). This also holds true for additional more specific values, beliefs, and experiences relevant during and in the aftermath of exogenous shocks (e.g., willingness to innovate, attitude to errors, trust in data, prioritization of employees’ well-being) (O12). BPM research should also investigate the extent to which these values and beliefs are sustained after a shock and deliver insights into their integration into a “new normal” culture to boost post-shock process performance. As an example, exogenous shocks can trigger increased mindfulness about the fit of existing processes in the context of external changes, which in turn boosts an organization’s intrinsic process-related flexibility (Baiyere et al. 2020). Finally, transferring approaches from organizational unlearning can prove useful in shaping a process culture open and willing to drop existing routines (C12) (Tsang and Zahra 2008). Organizational unlearning and similar approaches related to reassessing learned and established routines can also be beneficial in communicating and implementing future radical process changes independent of exogenous shocks (O11), e.g., in light of digital innovation (Mendling et al. 2020).

On top of the afore-mentioned implications regarding the core elements of BPM, we also pose that BPM research should explore specific shock-related capabilities. Current research has shown that transformative phenomena such as digitalization require novel BPM capabilities (Kerpedzhiev et al. 2020). Most likely, this holds true for exogenous shocks as well. Hence, more research is needed to identify which existing and additional BPM capabilities are required to cope with exogenous shocks across all phases successfully. To that end, the phase model presented in Fig. 2, together with the six core elements of BPM, form a matrix-like structure that may guide researchers in identifying new BPM capabilities related to exogenous shocks.

As an overarching insight, it has become evident that BPM and crisis management can considerably benefit from one another in manifold areas. In line with the propositions in the recent call for the establishment of process science (vom Brocke et al. 2021b), we pose that business processes can serve as a reasonable lens for understanding, analyzing, and managing organizational change induced by exogenous shocks. Therefore, we encourage the BPM, crisis management, and related research communities such as organizational resilience and high reliability organizations to evaluate specific opportunities for cross-discipline knowledge transfer but also to leverage corresponding synergies.

## Conclusion

In this research note, we set out to explore the intersection of BPM and exogenous shocks. Although exogenous shocks and related terms are extensively covered in other disciplines, they have not yet been addressed by BPM research, which has focused on other kinds of process change. However, since exogenous shocks can disrupt an organization’s context, strategy, and processes, they are highly relevant from a BPM perspective. Against this backdrop, we conceptualized the interplay of exogenous shocks and BPM in terms of their effects on overall process performance. Thereafter, we identified related challenges and opportunities for BPM and discussed these findings against the current BPM and related literature.

Like any other work, ours is beset with limitations that future research needs to be aware of. First, the presented challenges and opportunities were derived from the individual input of BPM researchers. While we cannot formally claim completeness and validity of our results, our approach is aligned with common standards and guidelines in conducting qualitative research. Nevertheless, future research should engage in exploring challenges and opportunities more rigorously (e.g., using exploratory interviews, case studies, or the Delphi method). Future research may also involve BPM practitioners, as this research note only covers the perspective of BPM scholars. Second, although we deliberately abstracted from specific exogenous shocks, we cannot exclude that our results are biased toward the COVID-19 pandemic, which was omnipresent when the research was conducted. While we believe that the presented challenges and opportunities are relevant beyond the COVID-19 pandemic, other shocks may entail new challenges or opportunities. For this reason, it is vital for future research to investigate the underlying mechanics of the effects presented in this research note and generalize them to allow for a comprehensive understanding of exogenous shocks. Finally, the relationship of exogenous shocks and overall process performance we outline in Sect. 4 was intended to serve as a foundation for the identification of challenges and opportunities. Consequently, it represents only typical effects of shocks on process performance discussed in the literature. Naturally, these effects including moderating factors need to be studied in more detail and backed by empirical works. This would also enable the identification of salient issues within the presented challenges and opportunities and empower researchers from disciplines related to crisis management to leverage possibilities for the integration of BPM-centered approaches.

By providing an initial conceptualization of the interplay of BPM and exogenous shocks as well as by presenting and discussing related challenges and opportunities, we hope our results stimulate a community-wide discussion on a hitherto neglected but highly relevant type of process change. In accordance with the topics discussed in Sect. 6, we call for more research located at the intersection of BPM and exogenous shocks. In light of currently aggravating global crises (e.g., climate crisis, rising geopolitical tensions), we believe that BPM research should not only understand the effects of exogenous shocks on business processes but also provide practitioners with guidance on how to mitigate shock-related challenges and leverage related opportunities throughout all phases of their management.
